# Clinical trials in vascular cognitive impairment following SPRINT-MIND: An international perspective

**DOI:** 10.1016/j.xcrm.2023.101089

**Published:** 2023-06-20

**Authors:** Fanny M. Elahi, Suvarna Alladi, Sandra E. Black, Jurgen A.H.R. Claassen, Charles DeCarli, Timothy M. Hughes, Justine Moonen, Nicholas M. Pajewski, Brittani R. Price, Claudia Satizabal, C. Elizabeth Shaaban, Nárlon C.B.S. Silva, Heather M. Snyder, Lukas Sveikata, Jeff D. Williamson, Frank J. Wolters, Atticus H. Hainsworth

**Affiliations:** 1 Friedman Brain Institute, Ronald M. Loeb Center for Alzheimer’s Disease, Icahn School of Medicine at Mount Sinai, New York, NY 10029, USA; 2 National Institute of Mental Health and Neurosciences, Bengaluru, Karnataka 560030, India; 3 Sunnybrook Health Sciences Centre, University of Toronto, Toronto, ON M4N 3M5, Canada; 4 Department of Geriatric Medicine and Donders Institute for Medical Neuroscience, Radboud University Medical Center, 6525 EN Nijmegen, the Netherlands; 5 Department of Neurology and Center for Neuroscience, University of California at Davis, Sacramento, CA 95817, USA; 6 Department of Internal Medicine, Wake Forest University School of Medicine, Winston-Salem, NC 27101, USA; 7 Alzheimer Center Amsterdam, Department of Neurology, Amsterdam Neuroscience, Vrije Universiteit Amsterdam, 1081 HZ Amsterdam, the Netherlands; 8 Department of Biostatistics and Data Science, Wake Forest University School of Medicine, Winston-Salem, NC 27154, USA; 9 Life Molecular Imaging, GmbH, Boston, MA 01209, USA; 10 Glenn Biggs Institute for Alzheimer’s and Neurodegenerative Diseases, Department of Population Health Sciences, UT Health San Antonio, San Antonio, TX 78229, USA; 11 Department of Epidemiology, School of Public Health, University of Pittsburgh, Pittsburgh, PA 15261, USA; 12 Djavad Mowafaghian Centre for Brain Health, Department of Physical Therapy, Faculty of Medicine, The University of British Columbia, Vancouver, BC V6T 1Z3, Canada; 13 Alzheimer’s Association, 225 N Michigan Avenue, Chicago, IL 60603, USA; 14 J.P. Kistler Stroke Research Center, Department of Neurology, Massachusetts General Hospital, Harvard Medical School, Boston, MA 02114, USA; 15 Division of Neurology, Department of Clinical Neurosciences, Geneva University Hospitals, 1205 Genève, Switzerland; 16 Institute of Cardiology, Lithuanian University of Health Sciences, Kaunas, Lithuania; 17 Department of Internal Medicine, Section on Gerontology and Geriatric Medicine, Wake Forest University School of Medicine, Winston-Salem, NC 27154, USA; 18 Departments of Epidemiology and Radiology & Nuclear Medicine, Erasmus MC, University Medical Centre Rotterdam, 3015 GD Rotterdam, the Netherlands; 19 Neurology, St George’s University Hospitals NHS Foundation Trust, London SW17 0QT, UK; 20 Molecular and Clinical Sciences Research Institute, St George’s University of London, London SW17 0RE, UK

**Keywords:** hypertension, blood pressure, dementia, cognitive impairment, clinical trials, aging, vascular disease, prevention

## Abstract

A large interventional trial, the Systolic Blood Pressure Intervention Trial sub-study termed Memory and Cognition in Decreased Hypertension (SPRINT-MIND), found reduced risk of cognitive impairment in older adults with intensive, relative to standard, blood-pressure-lowering targets (systolic BP < 120 vs. <140 mm Hg). In this perspective, we discuss key questions and make recommendations for clinical practice and for clinical trials, following SPRINT-MIND.

Future trials should embody cognitive endpoints appropriate to the participant group, ideally with adaptive designs that ensure robust answers for cognitive and cardiovascular endpoints. Reliable data from diverse populations, including the oldest-old (age > 80 years), will maximize external validity and global implementation of trial findings. New biomarkers will improve phenotyping to stratify patients to optimal treatments. Currently no antihypertensive drug class stands out for dementia risk reduction. Multi-domain interventions, incorporating lifestyle change (exercise, diet) alongside medications, may maximize global impact. Given the low cost and wide availability of antihypertensive drugs, intensive BP reduction may be a cost-effective means to reduce dementia risk in diverse, aging populations worldwide.

## Graphical abstract

**Figure:**
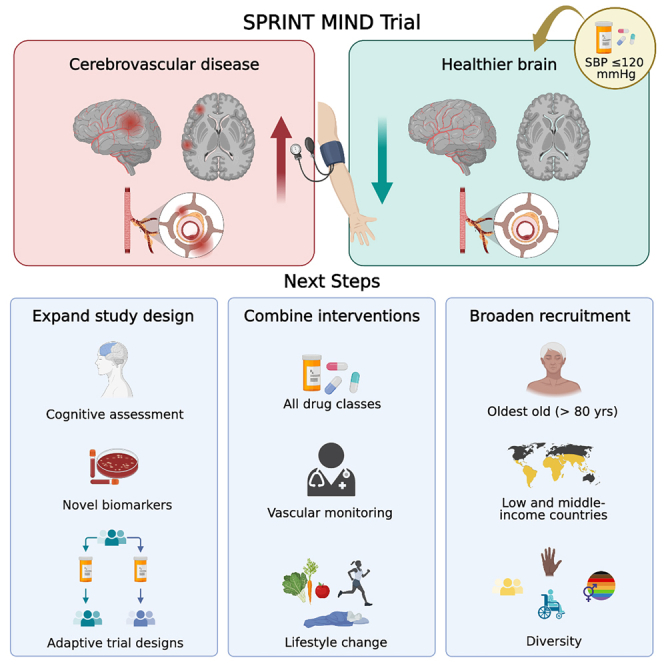

## Teaser

SPRINT-MIND was a landmark clinical trial, suggesting that intensive blood pressure lowering with cardiovascular drugs can ameliorate late-life cognitive impairment. Elahi et al. discuss challenges and opportunities for future clinical trials and for clinical management of older people at risk of dementia.

## Introduction

Vascular disease is now recognized as a major contributing factor in dementia, usually as a comorbid condition with Alzheimer’s disease (AD).^1^^,^^2^^,^^3^ Vascular risk factors contribute to cognitive impairment^4^ and are recognized risk factors for dementia.^5^ Acknowledging the major contributions of vascular disease to cognitive impairment has led to the concept of vascular contributions to cognitive impairment and dementia (VCID).^6^ The importance of VCID and the heterogeneity of dementia pathology^3^ has implications for prevention and precision medicine. This is especially important in diverse populations where ethnic differences affect the prevalence of vascular comorbidities.^7^^,^^8^

Recently observed declines in the age-specific incidence of dementia in North America and Europe have shown that dementia risk is modifiable, likely due to better cardiovascular risk management.^9^ While these trends temper the increasing burden of dementia, prevalence is still expected to rise substantially over the next decades,^9^^,^^10^ particularly in low- and middle-income countries (LMICs). Targeted treatments are therefore important, but few licensed drug treatments are available. For 20 years, these were limited to three acetylcholinesterase inhibitors and a non-selective *N*-methyl-d-aspartate (NMDA) receptor antagonist, all with modest clinical effects. Amyloid-depleting antibodies aducanumab and lecanemab were recently approved by the US Food and Drug Administration (FDA) for Alzheimer-type dementia.^11^^,^^12^ Given the sparse treatment options, therapeutic strategies for prevention of dementia are much needed. Treatment of vascular risk factors may offer novel therapeutic targets for dementia prevention and treatment.^13^^,^^14^

The Systolic Blood Pressure Intervention Trial sub-study termed Memory and Cognition in Decreased Hypertension (SPRINT-MIND) was a large multi-center study comparing intensive blood pressure (BP) lowering (target, systolic BP [SBP] <120 mm Hg) with a standard BP target (SBP < 140 mm Hg). There was significant 20%–30% reduction in risk for major cardiovascular events with intensive BP lowering (the primary outcome of the parent study, SPRINT).^15^^,^^16^ This led to early termination of the trial by the sponsor at 3.3 years, rather than the planned 6 years. The intensive intervention showed a null effect on the SPRINT-MIND primary endpoint of dementia diagnosis, possibly due to the reduced duration of the study. Nevertheless, intensive BP lowering significantly reduced mild cognitive impairment (MCI) and a combined adjudicated cognitive endpoint of MCI or probable dementia.^15^

These positive findings of a cognitive benefit in a large interventional trial are the basis for our emphasis on SPRINT-MIND in this perspective. Evidence for cognitive treatment benefits in the dementia field are few. That said, there have been several previous large trials in the area of BP lowering for cognitive benefit, previously reviewed.^17^ Individually, these trails were not decisive in demonstrating significant benefit in terms of reducing dementia risk.^17^ A recent meta-analysis of individual participant data from five large double-blinded studies, ADVANCE, PROGRESS, SHEP, SYST-EUR, and HYVET (data from N = 28,000 participants in total) detected a significant reduction in dementia risk with late-life BP lowering.^18^

A major strength of SPRINT was that it was not designed to test any particular drug class but rather the hypothesis that BP lowering affects end-organ dysfunction. The results were consistent with recent large meta-analyses of BP-lowering studies, concluding that the cognitive benefits of BP control are not strongly dependent on a specific class of medication.^19^^,^^20^ The results also demonstrated that generic, and therefore low-cost, formulations of standard BP-lowering drugs are adequate to achieve the cognitive preservation reported in SPRINT-MIND. Population modeling predicts that substantial global cardiovascular disease (CVD) prevention could be achieved with generic medications at an average cost of approximately US$1 per year.^21^ Furthermore, a recent review suggests that the majority of published studies on cost-effectiveness of hypertension interventions (both pharmaceutical only and combination programs) in LMIC demonstrate cost-effectiveness when evaluated based on cost per averted disability-adjusted life-year (DALY).^22^ Thus, intensive BP reduction may be a cost-effective means to reduce dementia risk in older people from diverse economic backgrounds worldwide.

There are caveats to this encouraging concept. Intensive BP lowering requires careful monitoring by physicians and other healthcare professionals. Also, despite clinical trial evidence to the contrary,^23^ many geriatricians, internists, and primary care physicians are reluctant to lower BP intensively in older persons, fearing to compromise cerebral blood flow (CBF). In addition, because most older patients—particularly vascular patients—have a high prevalence of multiple comorbidities (chronic renal disease, diabetes mellitus, pulmonary disease, rheumatologic and neurological conditions)^24^ and the attendant co-medications, there is reluctance to support intensive BP control requiring additional medication. Thus, managing prevention of VCID in older people will require a multifactorial approach. Finally, we cannot assume that a US-based treatment regimen (as in SPRINT-MIND) will translate straightforwardly to diverse populations in other global healthcare settings.

In this perspective, we review the implications of SPRINT-MIND for current clinical practice, and for future trials, by discussing 10 outstanding questions. Can SPRINT-MIND findings translate into frontline clinical practice? Do they apply in LMIC? Should inclusion criteria be broad or narrow? Who needs to be treated and when? Is cerebral hypoperfusion or orthostatic hypotension a concern? Are particular drug classes most beneficial? Would a combined approach including lifestyle interventions be preferable? Can genetics and biomarkers improve risk stratification? Should we include the oldest-old in future trials? Is it ethical to include a control group without intensive BP lowering? We offer suggestions for future trial design and practical implementation.

### SPRINT-MIND: Design and outcomes

#### Design

The parent trial SPRINT randomized participants to a cardiovascular drug regimen of either intensive or standard BP lowering, standard being the American Heart Association guideline goal at the time of trial initiation (SBP < 140 mm Hg). The primary hypothesis was that CVD event rates would be lower in the intensive arm. The design is summarized in Table 1.

**Table 1:** Overview of the SPRINT trial design

Sponsor	National Heart, Lung, and Blood Institute
Start date; completion date	October 2010; July 2016 (for primary outcome measure)
Primary outcome measures	number of participants with first occurrence of a myocardial infarction, acute coronary syndrome, stroke, heart failure, or CVD death
Secondary outcome measures	(1) Number of participants with all-cause mortality (2) Number of CKD Participants with at least 50% decline from baseline eGFR (3) Participants who developed end-stage renal disease (4) Number of patients with all-cause dementia. Basic Cognition Screening Battery; then, Extended Cognitive Assessment Battery plus the Functional Assessment Questionnaire for daily living skills. All data were adjudicated by a central panel of dementia experts. (5) Small Vessel Cerebral Ischemic Disease. Change in total white matter lesion volume from baseline; change in total brain volume from baseline
Study design	allocation: randomized intervention model: parallel assignment masking: single (outcomes assessor) primary purpose: treatment
Intervention	Arm 1: intensive control of SBP goal of SBP < 120 mm Hg. Use of once-daily antihypertensive agents was encouraged unless alternative frequency is indicated/necessary. One or more medications from the following classes: ACE inhibitors, ARBs, direct vasodilators, thiazide-type diuretics, loop diuretics, potassium-sparing diuretics, beta-blockers, sustained-release CCBs, alpha1-receptor blockers, sympatholytics arm 2: standard control of SBP goal of SBP < 140 mm Hg. Same medications as Arm 1
Actual enrolment	9,361 (target: 9,250)
Eligibility criteria	inclusion criteria: at least 50 years old, female/male SBP of: • 130–180 mm Hg on 0 or 1 medication • 130–170 mm Hg on up to two medications • 130–160 mm Hg on up to three medications • 130–150 mm Hg on up to four medications risk (one or more of the following): (1) Presence of clinical or subclinical cardiovascular disease, other than stroke (2) CKD, defined as eGFR 20–59 mL/min/1.73m2 (3) Framingham Risk Score for 10-year CVD risk ≥15% (4) Age greater than 75 years exclusion criteria include: • known secondary cause of hypertension, causing concern regarding safety of the protocol. • one-minute standing SBP <110 mm Hg. • proteinuria (within the past 6 months) • arm circumference too large or too small to allow accurate BP measurement • history of stroke (not cardioembolic or stenting), or cardiovascular event or procedure, or hospitalization for unstable angina within last 3 months, or diabetes mellitus • polycystic kidney disease; or glomerulonephritis, with immunosuppressive therapy; or end-stage renal disease • symptomatic heart failure within the past 6 months, or left ventricular ejection fraction <35% • living in the same household as a SPRINT participant

For full details of the SPRINT-MIND trial design, see ClinicalTrials.gov Identifier: NCT01206062 (from clinicaltrials.gov). CKD, chronic kidney disease; eGFR, estimated glomerular filtration rate.

#### Interventions

SPRINT was an open-label trial. Use of once-daily antihypertensive agents was encouraged unless alternative frequency was indicated/necessary. One or more medications from the classes listed in Table 1 were provided by the study for use in managing participants in both randomization groups. Preferred regimens included a thiazide diuretic (drug of choice, chlorthalidone), plus a calcium channel blockers (CCB) (drug of choice, amlodipine), plus an angiotensin-converting enzyme (ACE) inhibitor/angiotensin receptor blocker (ARB) (Table 1). The order in which agents were selected was left to the investigator. It was expected that many patients would need at least three antihypertensive drugs to achieve SBP < 120 mm Hg. Most (90%) of the medications used in SPRINT were generic drugs.

#### Outcomes

A total of 9,361 older Americans were enrolled, including 35.6% women, 30% African American, and 10.5% Hispanic, and 1,167 (12.5%) were aged 80 years or more at entry.^16^ Early termination of the trial (at 3.3 years) resulted in limited follow-up time to observe the development of dementia. Follow-up for cognitive and kidney outcomes continues during the post-intervention phase.

In terms of cognitive outcomes, intensive treatment did not lead to decreased risk of probable dementia over a median follow-up of 5.1 years, although it reduced the occurrence of MCI and a composite measure combining MCI or probable dementia (Table 2).^15^ In a non-random subgroup of participants that received comprehensive neuropsychological testing at each cognitive assessment, intensive treatment showed no difference on a composite measure of memory function, but slighter larger decreases on a composite measure of processing speed (driven by small differences on the Trail Making Test, Part B).^25^ Somewhat contrasting results were observed in an MRI sub-study. Intensive treatment was associated with a smaller increase in the volume of white matter lesions (WMLs), an MRI marker for cerebral small vessel disease (SVD),^26^ and with increased CBF.^27^^,^^28^^,^^29^ By contrast, intensive treatment was associated with larger decreases in total brain volume and hippocampal volume.^27^^,^^28^^,^^29^ Smaller increases in WML volume and larger decreases in total brain volume were similarly observed with intensive treatment in another large trial (ACCORD).^30^

**Table 2:** What SPRINT-MIND showed: Cognitive and MRI outcomes

	Intensive treatment	Standard treatment	–	–	–
Adjudicated cognitive impairment (N = 8563, median follow-up = 5.1 years)					
–	Cases per	Cases per	Hazard ratio	–	–
Outcome	1,000 person-years	1,000 person-years	(95% CI)^a^	p value	Reference
Probable dementia	7.2	8.6	0.83 (0.67–1.04)	0.10	Williamson et al.^15^
Mild cognitive impairment	14.6	18.3	0.81 (0.69–0.95)	0.007	
Composite of MCI or probable dementia	20.2	24.1	0.85 (0.74–0.97)	0.01	
Cognitive decline outcomes (N = 2921, median follow-up = 4.1 years)					
	Yearly	Yearly	Difference	–	–
Outcome	Slope (95% CI)^b^	Slope (95% CI)^b^	(95% CI)	p value	Reference
Memory domain^c^	−0.005	−0.001	−0.004	0.33	Rapp et al.^25^
	(−0.01 to 0.001)	(−0.006 to 0.005)	(−0.012 to 0.004)	–	
Processing domain^d^	−0.025	−0.015	−0.01	0.02	
	(−0.03 to −0.019)	(−0.021 to −0.009)	(−0.017 to −0.002)	–	
MRI outcomes (baseline N = 670, median follow-up = 4.0 years)					
	Mean change	Mean change	Difference	–	–
Outcome	(95% CI)^e^	(95% CI)^e^	(95% CI)	p value	Reference
White matter lesion volume, cm^3^	0.92	1.45	−0.54	<0.001	Williamson et al. 2019^15^; Nasrallah et al. 2021^28^; Dolui et al. 2022.^27^
	(0.69–1.14)	(1.21–1.70)	(-0.87 to −0.20)	–	
Total brain volume, cm^3^	−30.60	−26.9	−3.7	0.006	
	(−32.3 to −28.8)	(−28.8 to −24.9)	(−6.3 to −1.1)	–	
Hippocampal volume, cm^3^	−0.06	−0.02	−0.033	0.03	
	(−0.08 to −0.04)	(−0.05 to 0.00)	(−0.062 to −0.003)	–	
Whole brain CBF, mL/100 g/min	1.46	−0.84	2.3	0.02	
	(0.08–2.83)	(−2.30 to 0.61)	(0.30–4.30)	–	

a Intensive treatment versus standard treatment based on stratified Cox proportional hazards regression model.

b Yearly slope assuming a linear trend based on a linear mixed model.

c Includes the Logical Memory I and II, Modified Rey-Osterrieth Complex Figure (immediate recall), and the Hopkins Verbal Learning Test, Revised (delayed recall).

d Includes the Trail Making Test (parts A and B) and Digit Symbol Coding.

e For MRI outcomes, change estimates at 3.98 years post randomization based on a linear mixed model minimally adjusting for intracranial volume and days since randomization, including random effects for participant and imaging facility. Estimates for white matter lesion volume are based on a robust mixed model formulation given the skewed distribution of that outcome. Estimates for hippocampal volume and CBF also adjusted for age and sex.

### What are the lessons learned from SPRINT-MIND? What should be done differently in future trials?

#### Adaptive design

Early termination of SPRINT due to the success in reducing major cardiovascular outcomes of death, myocardial infarction, and stroke reduced the power to detect the impact of treatment on cognitive outcomes. This is compounded by gradual return of BP to pre-enrolment levels following trial completion.^31^ With hindsight, the design of SPRINT should have included an alternative design trigger that facilitated continued assessment of cognitive outcomes beyond the finding of a beneficial effect on CVD. Effects on cognitive function require longer-term follow-up than CVD endpoints. For this reason, it will be advantageous to include adaptive trial designs in future studies assessing cognitive impact, especially if there is CVD assessment in the trial. For example, study participants may be enrolled in an extended open-label treatment protocol to assess the impact of both short- and longer-term treatment on cognitive outcomes.

#### Additional endpoints

Other endpoints more sensitive to the impact of CVD on brain structure and function could be included, to support the biological plausibility of treatment efficacy. Such surrogate markers could include the burden of SVD and white matter microstructure. Additionally, assessment of potential intermediates and effect-modifying characteristics (pulse wave velocity, BP variability) may help to understand which BP lowering interventions are most efficacious, and in which patients.

Several recent prospective cohort studies (Diverse-VCID; MESA-MIND)^32^ were designed to examine pathways by which vascular risk factors contribute to cognitive decline in diverse groups of at-risk individuals. These studies will likely identify new biomarkers and intervention targets. MarkVCID^33^^,^^34^ and the HARNESS Initiative^35^ are consortia currently examining and harmonizing various blood-based and imaging outcome biomarkers to identify the best measures for clinical trials related to VCID. Advanced diffusion-based MRI techniques are increasingly being employed to image white matter microstructural injury.^35^^,^^36^

#### Greater diversity

SPRINT-MIND was a US-based study. Greater geographic diversity in trials will be advantageous, not only to understand intervention impacts in diverse populations^7^ but also because countries with high prevalence of hypertension stand to benefit most at the population level. This is critical in LMICs given their increasing dementia incidence. The PROGRESS study included some diversity^37^ but did not specifically target diverse individuals at highest risk for hypertension, such as African Americans. Risk stratification, therefore, should be considered along with diverse inclusion in designing future trials.

#### Lessons learned: Summary

The lessons learned from SPRINT-MIND include adaptive trial protocols; integration of cognitive outcomes in cardiovascular treatment trials; proposed lengthening of such trials for cognitive outcomes, possibly through open-label extension, when the primary cardiovascular outcomes are attained; increasing participant diversity; and risk stratification in study design. We welcome development of better vascular biomarkers for assessment of brain injury and for use as secondary measures in treatment trials.

### Is there a need for earlier interventions and longer trials?

Results from SPRINT-MIND emphasize the need for timely intervention, in line with the stronger link of mid-life CVD risk factors (especially BP) with dementia incidence, compared with late-life risk factors.^38^ Although in SPRINT-MIND only 3.3 years of treatment were needed to show cognitive benefit, it seems likely that benefits will increase with more prolonged treatment (5 years or more). Reductions in BP from mid-life onward, either through individualized or public health interventions, may therefore help to maintain cognitive health into old age.

Because dementia reflects end-organ damage to the brain, trials should focus on the inclusion of participants who are at risk and monitor events over longer periods of time. This has financial implications requiring commitment from governments and funders, particularly if trials are to be conducted in younger populations, where dementia incidence is low and cognitive testing can be hampered by ceiling effects. Sensitive and well-validated surrogate outcome measures may aid in detecting treatment effects in individuals prior to the onset of cognitive deficits. Detection of early cognitive impairment is a priority, with a focus on its prevention in the phase of subjective complaints (or even before). This requires increased public awareness about the benefits of treatment before the onset of cognitive impairment,^38^ which, in turn, has implications for developing methods for managing inclusion of trial populations. Recognition of VCID should be highlighted across different settings, including primary care and public health campaigns within the community.

#### Intervention timing: Summary

The optimal approach to dementia prevention through BP lowering starts in mid-life and will require individual as well as population interventions. Feasibility of individual participant trials will depend on prolonged duration of observation and treatment, and employment of more sensitive (possibly subclinical) outcome measures.

### Is orthostatic hypotension a concern?

Orthostatic hypotension (OH) refers to the acute fall in BP that results from standing up suddenly from a sitting or lying position. OH is often due to slowing of the autonomic reflexes that maintain adequate perfusion pressure and is frequently seen in older persons. A salient concern with OH is the risk of insufficient brain perfusion and possible loss of consciousness. There is a theoretical concern that intensive BP lowering may compromise CBF, and many medical personnel are apprehensive about intensive treatment at older ages. Observational evidence has suggested adverse outcomes of a lower BP, including cognitive decline and mortality.^39^ In patients with impaired CBF autoregulation, intensive BP lowering could indeed be a cause for concern; however, research in older people shows that, in fact, autoregulation remains largely intact with aging,^40^^,^^41^ even in persons with MCI and dementia.^42^ Measuring CBF before and after BP lowering in people with hypertension, including older adults, as a rule revealed no reductions in cerebral perfusion.^41^ This was confirmed in a recent systematic review, which included MCI and dementia.^23^ In SPRINT-MIND, intensive treatment was associated with a small but significant increase (4%) in whole-brain CBF (Table 2).^27^ Smaller studies confirm that there is no concern for a reduction in CBF with intensive BP lowering.^23^^,^^43^

In SPRINT, incidence of OH was not more common in the intensive BP-lowering group, nor was baseline OH associated with incidence of adverse events. There was more self-reported or clinician-reported syncope in the intensive group relative to the control group (3.5% vs. 2.4%)^44^ but with the caveat of possible bias due to “open-label” treatment allocation. There was no increase in electrolyte abnormalities, acute renal failure, or injurious falls in the intensive group. A careful examination of OH in SPRINT and meta-analysis of several randomized controlled trials (RCTs) of antihypertensive therapy concluded that “symptomless OH during hypertension treatment should not be viewed as a reason to down-titrate therapy even in the setting of a lower BP goal.”^45^^,^^46^ Limitations of these studies were that OH was measured only using the transition from sit to stand, which is less sensitive than supine to stand, and only at 1 min. Future studies should include more detailed measures of OH at initiation and discontinuation of antihypertensive therapy.

While SPRINT did not include persons with prevalent dementia, it is important to recall that concerns of autonomic dysfunction in dementia are mainly based on Lewy body dementia and Parkinson’s disease dementia, with limited evidence of autonomic dysfunction in AD and vascular dementia. In the NILVAD trial testing the CCB nilvadipine in AD patients,^47^ and in several smaller studies, there was also no evidence of increased risk of OH in patients with dementia, also with prolonged standing (up to 5 min).^42^

#### Orthostatic hypotension: Summary

For older people without dementia, OH appears not to be a major concern in BP lowering, and the prevalence of autonomic dysfunction is low (in view of physiological data from patients with AD, as well as SPRINT-MIND). High BP is itself an important cause of OH, and persons exhibiting OH may benefit from antihypertensive treatment. Overall, current evidence suggests that BP lowering does not lead to CBF reduction in older patients, with or without cognitive impairment or dementia.

### How to translate the SPRINT-MIND findings into a frontline clinical setting? Who needs to be treated, when do they need to be treated, and to what target?

From a clinical perspective, early recognition of vascular risk factors is key, as is the need to identify and prioritize treatment of risk factors with the largest health effect. For instance, the decrease in BP from baseline (the “delta”) may be more important for cognition than an absolute BP target (such as SBP < 120 mm Hg). Rather than one BP fits all, individuals with diverse risk factors, comorbidities, and biological characteristics may require different BP thresholds. After termination of SPRINT-MIND, SBP in the intensively treated group increased back toward previous guideline levels within 4–5 years.^31^ Not surprisingly, maintaining lower SBP, closer to 120 mm Hg, requires ongoing monitoring and management.

Despite the demonstrated benefits of BP lowering, mechanisms underpinning this remain to be elucidated. Understanding the molecular pathways modified will provide an opportunity to target these pathways, with a larger effect size. There remains an outstanding question of whether the benefits of BP lowering on cognition are mediated through neurodegenerative molecular dysfunctions, such as loss of neuro-glial proteostasis, or through a blood vessel delimited process. Implementation of molecular biomarkers in future trials will shed light on these important unknowns, with great relevance to preventive therapeutics for dementia.

Currently the only vascular and neurodegenerative risk stratification performed in clinical practice is through structural brain imaging, vital signs, and some basic laboratory tests. While more sensitive imaging and molecular biomarkers will improve clinical risk stratification, the feasibility of implementing these within large healthcare systems is a considerable challenge. Routine assessment of cerebral autoregulation, blood-brain barrier function, and neurovascular coupling, all important contributions from vascular dysfunction to cognitive impairment, are not currently feasible at scale in clinical practice. While such measurements are performed in research groups, thresholds and clinical interpretation at n-of-1 levels, needed for implementation in clinical practice, remain to be determined.

Implementing sensitive and specific vascular biomarkers that can be quantified non-invasively (for example, in blood) and are interpretable on an n-of-1 level will change clinical practice and prove instrumental for identifying persons at high risk and for guiding personalized, impactful interventions. Akin to their use in cancer and heart disease, biomarkers (imaging and molecular) in VCID can help to (1) stratify persons at increased risk of VCID who are most suited for interventions, (2) guide the selection and tailoring of the intervention to the individual (“n = 1 medicine”), (3) monitor the response to treatment, and (4) minimize side effects. The MarkVCID consortium is developing biomarkers of VCID for future clinical trials. MarkVCID initially evaluated 11 novel fluid^34^ and neuroimaging-based^33^ biomarkers of SVD, several of which progressed to the second round of clinical validations. Three novel fluid biomarkers for VCID were identified: plasma vascular endothelial growth factor (VEGF), placental growth factor (PlGF) and fibroblast growth factor (FGF)-2.^34^^,^^48^ In addition, CSF concentrations of PlGF and two endothelial inflammation markers (C3b and Bb measured from endothelial-derived extracellular vesicles) were deemed to be too early in development to be validated for clinical trials.^34^^,^^48^ These promising biomarkers continue to be investigated, with potential to give invaluable specificity for vascular disease. The initial neuroimaging-based candidate biomarkers include WML volume, WML growth/regression, peak width of skeletonized mean diffusivity, arteriolosclerosis, MRI free water, cerebrovascular reactivity, and optical coherence tomography angiography (OCTA) for retinal capillaries.^33^ From this list, WML volume and OCTA were eliminated from further validations in MarkVCID. Subcortical CBF^49^ and cerebral vasoreactivity^50^ have recently been used as outcome measures in clinical trials. Overall, vascular biomarkers have the potential to facilitate the early identification of SVD pathology and offer better monitoring of disease progression or intervention efficacy.

#### Translation to practice: Summary

Effective BP treatment for cognitive health is likely to require some clinical phenotyping for patient stratification. Better biomarkers, especially reliable, cost-effective, non-invasive biomarkers with proven specificity for VCID, could be implemented into clinical trials and eventually clinical practice. They will leverage risk stratification and fine-tuning of therapies. Plasma biomarkers, such as some investigated in MarkVCID, are promising candidates.

### Are SPRINT-MIND findings applicable in LMIC healthcare systems?

The burden of hypertension is rapidly rising in LMICs and the prevalence is higher compared with high-income countries (HICs). There is a wide treatment gap for hypertension and only a minority of hypertensives in LMICs receive treatment, due to low awareness, poor socioeconomic status, limited access to healthcare services, and poor treatment adherence.^51^ Therefore, there is a large population at risk of VCID, reflected in the higher prevalence of vascular dementia diagnoses in LMICs. Robust evidence for the benefits of BP lowering and reducing risk of VCID in this large vulnerable population is lacking. There is observational evidence regarding natural history of hypertension and other vascular diseases in LMICs, and some well-characterized longitudinal cohorts of persons with vascular disease have been established. The majority of trials examining vascular risk factor control on dementia risk—including SPRINT-MIND—are conducted in HICs and may not represent global diverse populations.^7^ While there is increasing government support and clinical research to reduce the burden of hypertension through public health programs^52^ and multi-center studies, there are still challenges for implementation of clinical trials comparable with SPRINT-MIND in LMICs^53^^,^^54^ (see Table 3). Nevertheless, management of hypertension does appear cost-effective even in resource-poor settings across various LMICs^22^ and therefore seems feasible to test as a dementia prevention strategy.

**Table 3:** Challenges for blood-pressure-lowering studies in LMICs

Category	Challenge
Risk stratification	well-characterized cohorts of individuals with hypertension and other vascular risk factors are needed across LMIC
Socio-demographics	differences in sociodemographic profiles between LMIC and HIC populations are likely to affect design and outcomes of clinical trials (Alladi et al., 2018)^8^
Life expectancy	life expectancy is lower in LMIC compared with HIC, changing the age profile of participants;, see World Bank data: https://data.worldbank.org/indicator/SP.DYN.LE00.IN
High comorbidity burden	the high burden of coexistent vascular risk factors, including untreated diabetes, metabolic syndrome, dietary factors, and cigarette smoking, must be considered in developing trial-specific cohorts
Cognitive assessment	trials that evaluate cognitive outcomes also require a uniform set of cognitive tests that are validated across diverse populations. Cognitive testing is challenging due to cultural, educational, and linguistic diversity. Harmonization efforts are underway to fill this gap, and validated neuropsychological batteries are now available in multiple languages and for different educational levels (Akinyemi et al.^53^; Iyer et al.)^54^
Biomarker standardization	imaging and plasma biomarkers of dementia and vascular disease also need to be standardized for diverse populations
Genetic studies	genetic factors, notably *APOE* genotype, may affect cognitive outcomes and should be systematically incorporated into study design
Infrastructure	infrastructure to implement trials is needed, including training of clinicians and researchers

#### The LMIC context: Summary

Clinical trials such as SPRINT-MIND demonstrating the impact of BP control have enormous potential to benefit cognitive outcomes in the context of LMICs. First, they may be fundamental to developing evidence to influence policy. Second, they may lead to population-based strategies to reduce the burden of VCID and dementia globally.

### Are particular classes of antihypertensive drugs more beneficial for brain health and cognitive outcomes?

**Figure 1:**
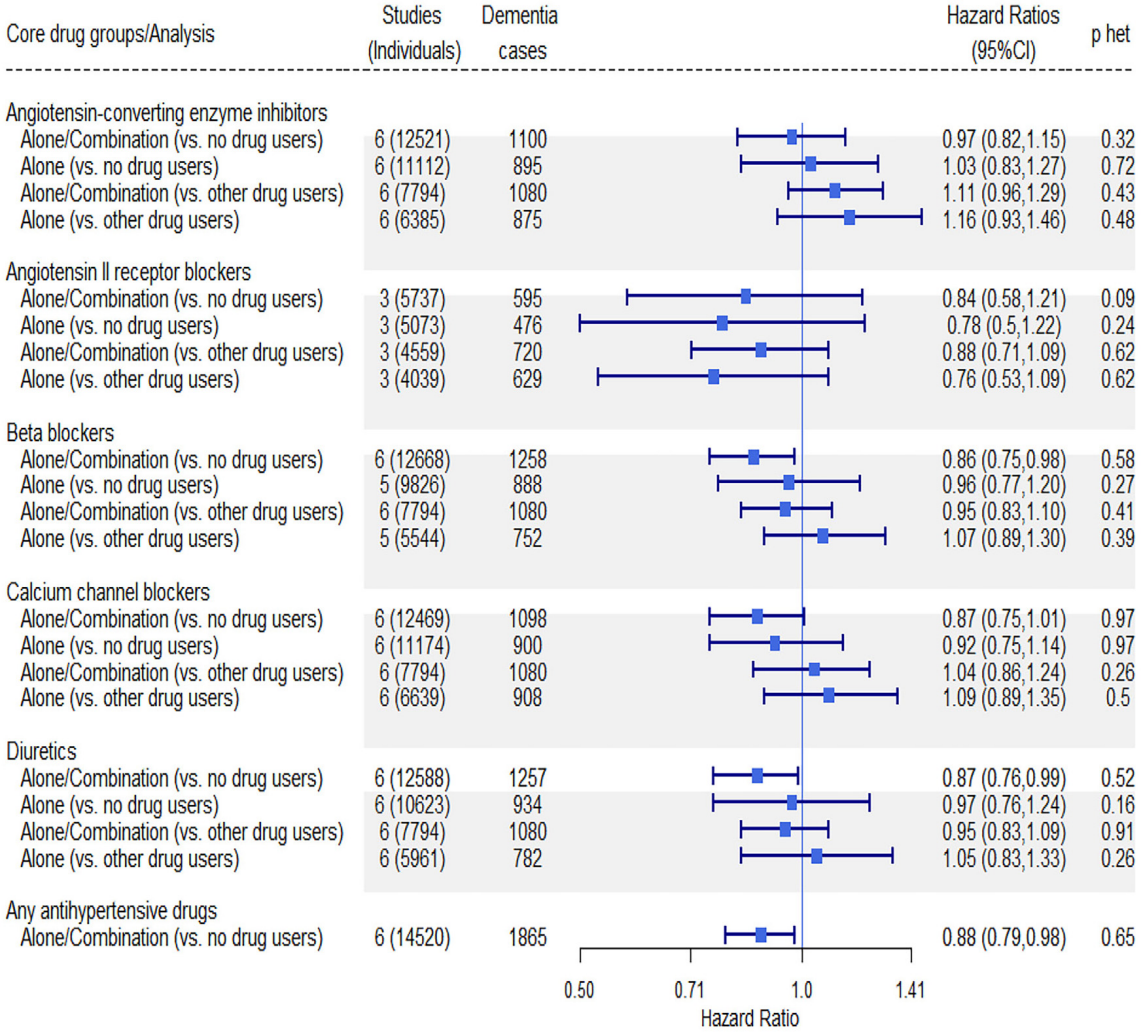
Associations of specific antihypertensive medication use with incident dementia in persons with high BP Pooled data from six population cohorts of prospectively recruited community-dwelling adults (N = 31,090). Horizontal symbols show hazard ratio (HR) (mean, 95% CI). The p values for heterogeneity (p-het) are listed. Adapted from Figure 1 of Ding et al.^19^ by permission from the publisher (Elsevier).

Meta-analyses of data from large observational cohorts^19^^,^^20^ support the notion that antihypertensive therapy may help prevent cognitive decline. One meta-analysis of individual patient data of six prospective community-based cohort studies, including over 31,000 participants, showed that antihypertensive treatment significantly reduced dementia risk but found no evidence for one drug class being more efficient than others (Figure 1).^19^ Another, larger meta-analysis including 27 studies and over 50,000 participants also found no consistent pattern to support any one antihypertensive drug class in cognitive decline or incident dementia.^20^

Single study evidence has suggested that CCBs or ARBs may particularly reduce dementia risk. The Systolic Hypertension in Europe (SYST-EUR) trial achieved the biggest reduction in incidence of dementia (by 50%) with a CCB,^55^ while trials with other drug types showed no or only modest benefits.^56^^,^^57^^,^^58^^,^^59^ An observational study suggested that healthy older adults and MCI patients taking ARBs had larger hippocampal volume, less atrophy, and better cognition compared with patients treated with ACE inhibitors,^60^ although these beneficial effects were not echoed by a trial in mild to moderate AD showing no cognitive benefits of ARB treatment.^58^ ARBs have a good blood-brain barrier penetration and may enhance the catabolism and clearance of Aβ. CCBs have a good blood-brain barrier penetration as well, have neuroprotective effects,^61^ and may reduce BP variability.^62^ The emerging importance of BP variability in subclinical SVD^63^ and dementia onset may reveal preferential class effects of certain BP-lowering medications over others. As the benefit of BP lowering is likely to reflect several causal factors (as in other diseases; e.g., chronic heart failure, chronic kidney disease) a holistic view of multiple pathways appears warranted.

Since SVD progression is likely to underlie dementia risk, a *post hoc* analysis of SPRINT-MIND drugs and SVD was carried out.^64^ Use of ACE inhibitors (β, −0.14 [95% confidence interval [CI], −0.23 to −0.04]; p = 0.002) and ARBs (β, −0.14 [95% CI, −0.263 to −0.05]; p = 0.003) had a small negative association with WML progression, while dihyropyridine CCBs showed mixed effects in logistic and linear models.^64^ These observational findings suggest there may be a modest class-specific pleiotropic effect of antihypertensive therapy on SVD progression.

#### Particular drug classes: Summary

Currently there is no convincing evidence that one antihypertensive drug class affords a larger reduction of dementia risk over another. These findings support clinical freedom in the selection of type of antihypertensive drugs to achieve BP goals. The possible protective effects of antihypertensive drugs in prodromal stages of neurodegeneration merit further exploration in prospective outcome-based studies.

### Should a VCID trial recruit the oldest-old (age 80+ years)?

**Figure 2:**
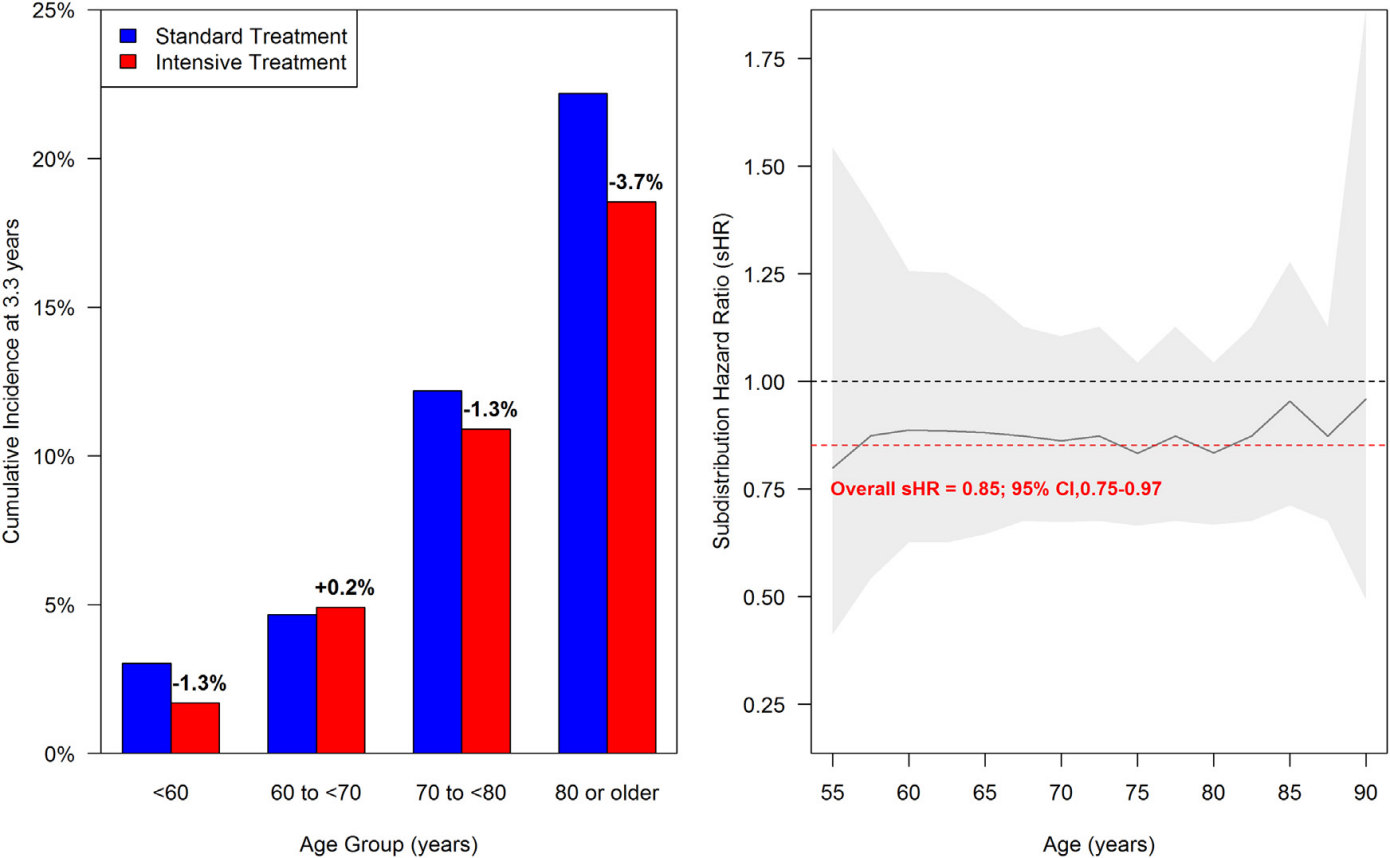
Risk reduction across the age range in SPRINT-MIND The left panel shows the cumulative incidence of cognitive impairment (a composite outcome of MCI or probable dementia) by age in SPRINT-MIND comparing intensive with standard treatment, accounting for the competing risk of death. The absolute risk reduction is greater in older adults (age 70 years or older), likely due to higher event rates. The right panel shows estimated overall and age-specific sub-distribution HR from a Fine-Gray competing risks regression model for cognitive impairment (same composite outcome) in SPRINT-MIND comparing intensive with standard treatment. Relative risk reduction is quite consistent across the age range. Shaded areas denote 95% CIs. Overall subdistribution Hazard Ratio (sHR) = 0.85, 95% CI = 0.75–0.97 (N.M.P., J.D.W., unpublished data).

The significant treatment effect detected in SPRINT-MIND was particularly evident in older participants (age > 70 years), although this positive finding may be driven by higher event rates in older people (Figure 2). Older adults without pre-existing MCI did well in SPRINT-MIND, and their overall adverse event rate did not differ between the treatment groups. This included careful monitoring of kidney function. Specifically, most cases of incident MCI or dementia (and most of the risk reduction) occurred in patients aged >75 years,^15^^,^^65^ supporting the safety of this intervention in older people. We speculate that, in older patients at risk of dementia, cardiovascular events (stroke, myocardial infarction, vascular surgery, or other cardiovascular interventions) may trigger delirium, and this in turn could progress to cognitive decline.^66^^,^^67^ Prevention of cardiovascular events may therefore contribute to prevention of dementia in this indirect pathway. Prevalence of hypertension, as well as incidence of CVD and dementia, is highest in older adult populations, so treatment is especially important for them.

#### The oldest-old: Summary

Trials of VCID should be designed to include the oldest-old. This age group may be instrumental for detection of treatment effects of intensive BP lowering.

### Should inclusion criteria be broad or narrow?

More crudely, should we lump or split? Lack of external validity of clinical trials can hamper the transportability of clinical trial results to practice. The perception by physicians that certain patient groups are under-represented in research studies is an important cause for under-use of effective treatments. Hence, there is a need to diversify the pool of individuals included in trials. Beneficial effects of BP lowering on cognition may be dependent on patient characteristics (race, comorbidities, cognitive status) or setting (primary care, memory, or stroke clinic). A typical older adult at risk of dementia has multiple comorbidities, all potentially modifying treatment efficacy. Hence larger samples are needed to confirm benefit in those most affected. In addition to age, sex, education, and *APOE-ε4* status, trajectories of disease progression may differ by race/ethnicity and respond differently to treatment.^68^ Broad and proportional inclusion criteria would potentially avoid replicating findings in different populations. It may be more efficient to use the established machinery of a large trial (recruitment resources, trained staff, equipment, start-up funding) to address a question of diversity than to launch entirely new studies.

Nonetheless, in view of anticipated clinical benefit, there are valid reasons to exclude individuals who are not at risk for the outcome measure of interest. Examples include those with limited life expectancy (such as nursing home residents) or healthy young persons who have no probability of contributing data to the trial outcomes. Inclusion of participants with high BP or evidence of cerebral SVD at baseline will increase trial power (analogous to screening patients for β-amyloid-positive status for inclusion in trials of amyloid lowering therapy).

Several counter-arguments complicate this strategy. First, benefits of BP lowering on CVD outcomes are clear in persons within the normal range of BP,^69^ supporting potential benefits in a wider population at risk of cognitive decline. Second, the need for early intervention favors inclusion of individuals before symptoms of vascular disease become manifest. With the advent of blood tests for AD markers (Aβ peptides; ptau-181, 217, or 231), we may be able to detect nascent AD pathology, alongside vascular risk, in individuals while they are still cognitively normal. Third, randomized assessment of treatment efficacy may not be feasible or ethical in those with other strong indications for treatment (such as acute cardiovascular events). Broad inclusivity in the design of forthcoming trials will benefit external validity and facilitate subgroup analyses of diverse clinical profiles and racial/ethnic background. SPRINT-MIND did not detect differences between pre-specified ethnic subgroups and was not powered to do so.

#### Inclusion criteria: Summary

There is a need to diversify the pool of individuals included in clinical trials. Diversification based on age, sex, education, and *APOE-ε4* status, alongside race, is critical as these factors may influence treatment response. Recruitment strategies should carefully consider the selection of participants in whom treatment efficacy can be demonstrated during the course of the trial. These should be weighed against effects of restricted inclusion on external validity and consequent lack of implementation of trial findings in routine clinical practice.

### Would a combined approach that includes lifestyle (not just drugs) be more effective?

In high-risk populations, an intervention based on tailored exercise alone may be beneficial for SVD and cognitive outcomes, although the available data are mixed.^70^^,^^71^ Such lifestyle interventions may be enhanced by expanding to multi-domain approaches (physical activity, diet, cognitive engagement, and vascular risk factor management)^72^ and could potentially maximize the benefits of pharmacological interventions. Current guidelines for management of hypertension already favor such a combined approach,^73^ and this could be extended to brain-related outcomes.

For individuals with cognitive impairment and CVD risk factors, a multi-domain intervention may improve executive function, memory, and learning, with additional reduction in BP.^74^ In a small study of participants who already had an AD dementia diagnosis, a multi-domain intervention was associated with reduced WML progression.^75^ Among dementia-free older adults, a much larger trial of a multi-domain intervention (PreDIVA) showed no difference in all-cause dementia but detected a positive effect on non-AD dementia favoring the intervention group.^76^

Beyond direct effects on cognition, exercise interventions can improve other determinants of cognitive function, such as mood regulation and physical mobility,^77^^,^^78^ while pharmacological treatment could exert specific effects in managing chronic illness and reducing overall risk. This is important, as lifestyle interventions alone did not lead to significant changes in CVD risk markers in the FINGER trial (BP, serum total cholesterol, fasting plasma glucose).^72^

Implementation and long-term sustainability of combined interventions may be challenging, especially in ethnically diverse populations, due to differences in dietary patterns, socioeconomic status, geographical location, and accessibility. The ongoing World-Wide FINGERS (WW-FINGERS) trial (including its US-based component POINTER) will be critical, as it addresses the implementation barriers in diverse populations. WW-FINGERS is testing a multifactorial intervention to reduce the risk for cognitive decline, based on the original FINGERS trial (based in Finland),^72^ which showed cognitive benefit of a multifactorial intervention. General recommendations for multi-domain trials were reviewed in an expert perspective^79^).

#### Combined approaches: Summary

With the success of SPRINT-MIND and growing evidence for the efficacy of lifestyle interventions, a combined approach seems pragmatic in dementia prevention. This multifactorial approach holds promise for additive effects on brain function, ultimately reducing dementia risk. Significant effort and funding will be necessary to perform definitive trials of these combined approaches, considering the complex implementation logistics and long-term sustainability.

### Is it unethical to include a control group without intensive BP lowering in future trials?

This is a challenging question. On one hand, a clinical trial should have a control group, and a randomization procedure to assign participants either to intervention or to control treatment. On the other hand, the beneficial effects of intensive BP lowering are clear in terms of mortality and major CVD (as shown by the main outcomes of SPRINT^16^^,^^44^). Hence investigators (and ethical review boards) may be reluctant to have participants assigned to an untreated control group. A control group treated with the national, accepted standard of care is more acceptable.

#### Control group: Summary

We agreed that it would be unethical to use a no-treatment control group and that intensive BP treatment can be appropriately compared with a control group treated with standard of care.

## Concluding remarks

Hindsight gets paradoxically clearer with time. SPRINT-MIND gave justified optimism for future cognitive trials. Since 2010, when SPRINT opened, we have learned a lot from SPRINT and other BP-lowering studies. There are numerous points where a future trial would be designed differently. We offer the following take-home messages. (1) Intensive BP lowering with cardiovascular medicines can achieve substantial decline in MCI/dementia risk. This is a foundation to build on. (2) In real-world clinical management, there is a need for early intervention, before symptoms of cognitive decline become manifest. (3) Future study design should include flexibility to adapt to early termination, while maintaining statistical power to determine effects on cognitive endpoints (including dementia incidence). (4) Dementia biomarkers are a flourishing field. Improved biomarkers will provide better, quantifiable trial endpoints and also greater understanding of physiological effects and risk mediators. (5) Researchers should be encouraged to work toward local and global generalizability, across socioeconomic and ethnic spectra. This is already in progress in WW-FINGERS. (6) Future studies should investigate specific mechanistic targets and whether there are antihypertensive drug classes that afford the greatest cognitive protection. (7) Future trials should consider combined interventions, with medication alongside lifestyle change (e.g., exercise, diet, cognitive training, social stimulation, vascular risk monitoring).
